# Polyphenol-functionalized silver nanoparticles promote differential remineralization and reinforcement of demineralized dentin

**DOI:** 10.1590/1678-7765-2025-0848

**Published:** 2026-05-01

**Authors:** Tattiana Enrich, Santiago González-López, Alejandro B. Rodríguez-Navarro, María V. Bolaños-Carmona, Carolina Cifuentes-Jiménez

**Affiliations:** 1 Universidad de Granada Facultad de Odontología Departamento de Odontología Operatoria Granada España Universidad de Granada, Facultad de Odontología, Departamento de Odontología Operatoria, Campus Universitario de Cartuja, Granada, España.; 2 Universidad de Granada Facultad de Ciencias Departamento de Mineralogía y Petrología Granada España Universidad de Granada, Facultad de Ciencias, Departamento de Mineralogía y Petrología, Granada, España.; 3 Universidad de Granada Facultad de Odontología Departamento de Odontología Pediátrica Granada España Universidad de Granada, Facultad de Odontología, Departamento de Odontología Pediátrica, Campus Universitario de Cartuja, Granada, España.

**Keywords:** Dental caries, Metal nanoparticles, Tooth remineralization, Crosslinking reagents, Flexural strength

## Abstract

Silver nanoparticles (AgNPs) have gained relevance in restorative dentistry due to their antimicrobial and remineralizing properties. However, the role of AgNPs functionalized with different polyphenols in dentin remineralization remains insufficiently understood.

**Objectives:**

This study aimed to synthesize and characterize AgNPs functionalized with crosslinking polyphenols extracted from grape seed extract (GSE) and green tea leaves (GT), and to evaluate their biocompatibility and their effects on the mechanical and physicochemical properties of demineralized dentin.

**Methodology:**

AgNPs were characterized by TEM, XRD, TGA, and zeta potential analysis to determine particle morphology, crystalline structure, chemical composition, and surface charge. Biocompatibility was assessed using fibroblast cytotoxicity assays. In total, 40 human mid-coronal dentin specimens were randomly assigned to four groups (n=10): Control- (sound dentin), Control+ (pH-cycled dentin without treatment), AgGSE (pH-cycled + AgGSE, 1 min), and AgGT (pH-cycled + AgGT, 1 min). Treated dentin was analyzed by ATR-FTIR, XRD, TGA, and three-point bending tests.

**Results:**

Both AgNPs were successfully synthesized and exhibited high biocompatibility. AgGSE demonstrated greater dentin matrix interaction, resulting in significantly higher flexural strength (p≤0.001). In contrast, AgGT induced pronounced remineralization with increased PO_4_/Amide I ratio (p=0.003) and CO_3_/PO_4_ ratios (p=0.007 by TGA; p=0.03 by ATR-FTIR), consistent with carbonated apatite deposition.

**Conclusions:**

Green-synthesized AgNPs functionalized with GT or GSE promoted reinforcement and remineralization of demineralized dentin through distinct mechanisms of action. These findings highlight their potential as biocompatible agents for dental therapies.

## Graphical abstract

**Figure:**
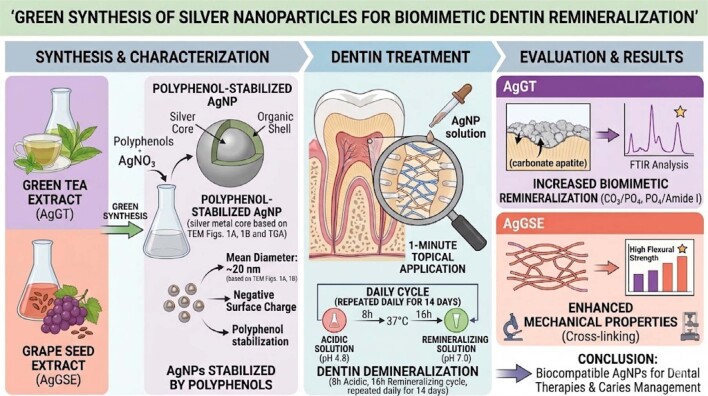

## Introduction

Dental caries is one of the most prevalent diseases worldwide. In 2019, an estimated 3.09 billion people had untreated caries in permanent teeth, representing a prevalence of 46.07% of the global population.^1^ This disease constitutes a major public health problem with a significant economic burden worldwide. Additionally, it has important psychosocial impacts, directly affecting patients’ quality of life and social interactions. Consequently, strategies for the prevention and treatment of dental caries remain a major focus of research. Over the past 20 years, investigations have increasingly focused on the development of nanoparticles (NPs) due to their unique physicochemical and biological properties, which may offer advantages over conventional dental therapies.^2,3^ Furthermore, NPs exhibiting simultaneous remineralizing, antimicrobial, and matrix-stabilizing properties are particularly promising for caries management, as they can inhibit bacterial growth while promoting collagen preservation and mineral deposition.^3,4^

Silver nanoparticles (AgNPs) have attracted significant attention in dentistry due to their broad-spectrum antimicrobial properties and potential to prevent tooth demineralization.^5^ The green synthesis of AgNPs using plant-derived extracts as reducing and stabilizing agents provides a cost-effective and environmentally sustainable alternative to conventional chemical methods, avoiding toxic by-products and enhancing biocompatibility.^7,8^ The incorporation of polyphenolic crosslinking agents further reinforces the dentin organic matrix, which is crucial because collagen integrity directly influences mineral deposition and mechanical stability.^9,10^

Grape seed extract (GSE) and green tea leaf extract (GT) are rich sources of polyphenolic compounds with proven antioxidant, antimicrobial, and matrix-stabilizing properties relevant to dental applications.^9,11^ GSE is particularly abundant in proanthocyanidins, which can effectively cross-link collagen fibers and enhance dentin mechanical properties.^9,12^ GT contains high levels of catechins, especially epigallocatechin-3-gallate (EGCG), known for superior antioxidant activity and collagen stabilization.^9,13^ These polyphenols serve not only as reducing and capping agents during AgNP synthesis but also as natural cross-linkers that strengthen the dentin organic matrix and improve resistance to enzymatic degradation.^9,14^

Given that the balance between demineralization and remineralization plays a central role in caries pathogenesis, biomaterials capable of simultaneously inhibiting demineralization, enhancing remineralization, and strengthening the collagen matrix represent a promising strategy for caries management.^15,16^ Although AgNPs synthesized from GSE or GT have been individually investigated for their biocompatibility and potent antibacterial/antibiofilm (GT) and antibacterial/antifungal (GSE) activities,^8,14,17,18^ no study has systematically compared their effects on demineralized dentin, particularly regarding mineral composition changes and their correlation with mechanical properties.

Therefore, this study aimed to synthesize and characterize AgNPs functionalized with polyphenols from *Vitis vinifera* grape seed extract (AgGSE) and *Camellia sinensis* green tea leaf extract (AgGT), and to comprehensively evaluate their differential effects on demineralized dentin. The novelty of this comparative study lies in the systematic analysis of how different polyphenolic functionalization affects not only the synthesis and properties of AgNPs but also their interactions with the dentin matrix at the molecular level. Understanding these structure-function relationships is essential for developing targeted therapeutic agents capable of addressing the multifactorial nature of dental caries through complementary mechanisms.

Synthesized AgNPs were characterized using transmission electron microscopy (TEM), X-ray diffraction (XRD), thermogravimetry (TGA), and zeta potential measurements. Biocompatibility was assessed through direct cytotoxicity assays on fibroblasts. The effects of AgNPs on demineralized dentin were evaluated using ATR-FTIR spectroscopy, TGA, XRD, and three-point bending tests to determine mineral composition, crystallinity, and mechanical properties. The following null hypotheses were tested: i) there are no differences in physicochemical characteristics between AgGSE and AgGT nanoparticles; ii) neither AgGSE nor AgGT demonstrate acceptable biocompatibility; and iii) AgGSE and AgGT do not produce different effects on the mineral composition or biomechanical properties of demineralized dentin.

## Methodology

### Biosynthesis and characterization of NPs

#### Materials and reagents

This study was approved by the Local Ethics Committee of the University of Granada (#1896-2020).

All chemical reagents, including silver nitrate (AgNO_3_), calcium chloride (CaCl_2_), sodium dihydrogen phosphate (NaH_2_PO_4_), acetic acid, and potassium chloride (KCl), were supplied by Sigma-Aldrich with >99.0% purity. Grape seed extract derived from *Vitis vinifera* (95% proanthocyanidin) was obtained from Infisa – Instituto Fitológico s.l. (Barcelona, Spain, batch nº S210296). Organic green tea (*Camellia sinensis*) leaves were purchased from Granadiet (Ogíjares, Spain, batch nº 224). All plant materials were stored in dark, dry conditions at room temperature until use.

#### Green synthesis of silver nanoparticles

AgGT nanoparticles were synthesized following a modified protocol based on Rolim et al. (2019). Briefly, 0.5 g of green tea powder was dispersed in 100 mL of Milli-Q water and heated at 70 °C under constant magnetic stirring (300 rpm) for 1 h. The resulting brown extract was filtered through a 0.1 μm membrane filter. For nanoparticle synthesis, 100 mL of freshly prepared 0.1 M AgNO_3_ solution was added dropwise (1 mL/min) to the tea extract under continuous stirring at room temperature (23±2°C). The solution immediately changed from yellow to yellow-gray (Figure S1), indicating the reduction of Ag^+^ ions to metallic Ag^0^ and formation of colloidal silver nanoparticles. The reaction mixture was maintained under stirring for 48 h at 23°C±2°C in darkness to ensure complete reduction and stabilization. The resulting colloidal suspension was centrifuged at 10,000 rpm for 10 min, and the supernatant discarded. The precipitated AgNPs were washed three times with Milli-Q water to remove unreacted silver ions and organic impurities, then dried at 37 °C for 24 h and ground to obtain a fine powder.

AgGSE nanoparticles were synthesized according to the method described by Ping, et al.^29^ (2018), with minor modifications. A stock solution was prepared by dissolving 0.5 g of GSE powder in 100 mL of Milli-Q water under magnetic stirring for 30 min at room temperature. Silver nanoparticle formation was initiated by dropwise addition (1 mL/min) of 100 mL of 0.1 M AgNO_3_ solution to the GSE solution under constant stirring. The solution immediately changed from light-brown to dark-brown (Figure S1). The reaction proceeded for 48 h at 23±2°C under continuous stirring in darkness. The final colloidal solution was centrifuged at 10,000 rpm for 15 min, and the precipitate washed three times with Milli-Q water to remove residual reactants. The purified nanoparticles were dried at 37°C for 24 h and ground to obtain a homogeneous powder.

#### Physicochemical characterization

Particle size, morphology, and distribution of synthesized AgNPs were determined using transmission electron microscopy (TEM) (Zeiss LIBRA 120 PLUS TEM, Carl Zeiss, Germany). Crystalline phase identification was performed using X-ray diffraction (XRD) with an X’Pert Pro diffractometer (PANalytical, The Netherlands) using CuKα radiation. The surface charge and colloidal stability of the AgNPs in aqueous suspension were evaluated through zeta potential measurements using a Zeta-check analyzer (Particle Metrix, Germany). Thermogravimetric analysis (TGA) was conducted using a TGA/DSC1 system (Mettler-Toledo, Switzerland) to determine the organic content and thermal stability of the functionalized nanoparticles. The organic matter content was calculated as the percentage weight loss between 200 °C and 600 °C (%OM loss), corresponding to the decomposition of organic compounds adsorbed on the nanoparticle surface.

For each formulation, TEM analysis and zeta potential measurements were performed on three independently prepared nanoparticle batches (5 mL aqueous dispersions each). XRD and TGA analyses were also conducted in triplicate using ~20 mg samples per nanoparticle type. For XRD, the average crystallite size of apatite crystals was calculated using the 002 reflection and the Scherrer equation. All physicochemical measurements were performed by a blinded operator. Data from replicate runs were averaged to ensure precision and statistical reliability.

## Cytotoxicity assay

### Cell culture and sample preparation

Mouse 3T3-L1 fibroblasts (ECACC 86052701, batch: 2618) were obtained from the CIC Culture Collection of the University of Granada (#1896-2020). Cells were cultured in Dulbecco’s Modified Eagle’s Medium (DMEM) supplemented with 2 mM glutamine, 10% calf serum (CS), and 1% penicillin-streptomycin solution (100 U/mL penicillin and 100 μg/mL streptomycin). Cultures were maintained at 37°C in a humidified atmosphere containing 5% CO_2_ and 95% air for 48 h.

AgGSE and AgGT nanoparticle stock solutions were prepared at 1 mg/mL in sterile Milli-Q water and sonicated for 10 min to ensure complete dispersion. Stock solutions were filter-sterilized through 0.22 μm cellulose acetate filters and stored at 4°C. Working concentrations (10, 50, 100, 200, and 300 μg/mL) were prepared by serial dilution in complete DMEM immediately before use. The concentration range was selected based on previous studies demonstrating that silver nanoparticles show biological activity within this range, with concentrations above 100 μg/mL potentially causing cytotoxic effects, while lower concentrations (10–50 μg/mL) are typically considered biocompatible for dental applications.^17,18^ Each experimental condition was tested in technical triplicates (3 wells per concentration) with 16 readings per well to ensure measurement precision and statistical reliability.

### Cell viability assessment

Cell viability was determined using the MTS [3 - (4,5-dimethylthiazol-2-yl) -5 - (3-carboxymethoxyphenyl) – 2 - (4-sulfophenyl) - 2H-tetrazolium] reduction assay (CellTiter 96^®^ Aqueous, Promega Corporation, USA). Briefly, 3T3 fibroblasts were seeded in 96-well culture plates (Nunc GMBH & Co. KG, Germany) at a density of 1×10^4^ cells per well in 100 μL of complete DMEM. Plates were incubated for 24 h at 37°C in 5% CO_2_ to allow cell attachment and exponential growth. After 24 h, the medium was carefully removed and replaced with 100 μL of fresh DMEM containing the test concentrations of AgGSE and AgGT (10–300 μg/mL). Each concentration was tested in triplicate (3 wells per condition), and cells cultured in DMEM without nanoparticles were included as a 100% viability control. After 24 h of incubation, 20 μL MTS reagent was added to each well and incubated for 4 h. Absorbance was measured at 490 nm using an Infinite 200 PRO Microplate Reader (Tecan Trading AG, Switzerland). In total, 16 readings per well were taken and averaged to provide a single, highly accurate absorbance value, and the mean absorbance of the control group was used to estimate 100% cellular viability.

## Preparation of demineralized dentin and application of nanoparticles

A total of 10 sound human third molars were collected for orthodontic or surgical reasons from patients aged 18–35 years after obtaining informed consent. Collected teeth were cleaned and stored in 0.1% thymol solution at 4 °C until preparation. Forty dentin beam specimens (6 × 1 × 1 mm^3^) were obtained from mid-coronal dentin using a diamond cut-off wheel (MOD 13, Struers, Denmark) on a precision cutting machine (Accutom 50, Struers, Denmark) under water cooling. Each specimen was rinsed with deionized water and ultrasonicated for 30 min to remove cutting debris. Complete enamel removal, uniform dentin surface exposure, and absence of microcracks were verified using stereomicroscopy (SZ-TP, Olympus, Japan) at 20× magnification. Only specimens meeting these criteria were included. Specimens were randomly assigned to four groups (n=10): Control- (sound dentin), Control+ (pH-cycled dentin without treatment), AgGSE (pH-cycled dentin treated with AgGSE for 1 min), and AgGT (pH-cycled dentin treated with AgGT for 1 min). Control- specimens were stored in deionized water at room temperature throughout the experiment to serve as baseline reference.

Artificial carious lesions were induced using a 14-day pH-cycling model, simulating the dynamic demineralization and remineralization of carious lesions.^19^ Each specimen was immersed in 1 mL of demineralizing solution (2.2 mM CaCl_2_, 2 mM NaH_2_PO_4_, and 50 mM acetic acid, adjusted to a pH of 4.8) for 8 h, followed by 16 h in 1 mL of remineralizing solution (1.5 mM CaCl_2_, 0.9 mM NaH_2_PO_4_, and 0.15 M KCl, adjusted to a pH of 7.0) at 37°C without agitation. Fresh solutions were prepared and renewed every 24 h.

After pH-cycling, specimens from Control+ were stored in deionized water at room temperature. For experimental groups, treatment solutions were prepared by dispersing 1% (w/v) of each AgNP powder (AgGSE and AgGT) in Milli-Q water using ultrasonication for 10 min. The treatment solution pH was 6.5. Specimens from AgGSE and AgGT groups were individually immersed in 1 mL of their respective solution for 1 min under gentle stirring (100 rpm) to ensure uniform contact. After treatment, specimens were immediately rinsed with 1 mL Milli-Q water for 1 min under gentle agitation to remove excess nanoparticles and prevent aggregation on the surface.^20-22^

## Analysis of treated dentin samples

### Infrared spectroscopy (ATR-FTIR)

Ten dentin specimens per group were analyzed using a Fourier Transform Infrared (FTIR) spectrometer (JASCO 6200, JASCO, Japan) with a diamond ATR accessory (ATR Pro ONE, JASCO, Japan). Spectra were recorded at 2 cm^-1^ resolution and 64 scans in a spectral range of 400–4000 cm^-1^ in absorbtion mode.^23^ From the peak area, the following compositional parameters were estimated to determine dentin chemical properties: the relative mineral to organic matrix ratio (PO_4_/Amide I) determined as the ratio of the main phosphate (ν_1_/ν_3_ PO_4_; 900–1200 cm^-1^) to the Amide I band area ratio (1590–1710 cm^-1^) and the amount of carbonate substituted in the mineral (CO_3_/PO_4_) estimated as the ratio of the carbonate band at 870 cm^-1^ to the main phosphate band.^24,25^

### X-ray diffraction (XRD)

Specimens were analyzed by XRD with an X’Pert Pro diffractometer (PANalytical, Almelo, Netherlands) using CuKα radiation. Scans were acquired between 30° and 70° (2θ values), with a step size of 0.0042°, and integration time of 5.08 s. The average crystallite size (D) of apatite crystals was calculated using XPowderX software and the Scherrer equation: D = Kλ / β cos θ; where K is a constant that varies on the crystalline habit and is chosen as 0.9 for elongated crystals of apatite, λ is the wavelength CuKα radiation (λ = 1.5406 Å), β is the full width at half maximum (FWHM) in radians of the 002 and 310-apatite diffraction peaks, and θ is the diffraction angle for this reflection (25.9° and 39°, respectively).

### Thermogravimetry (TGA)

Samples were prepared for thermal analysis by careful grinding using a mortar and pestle to obtain homogeneous powder. Sample mass was standardized at 10 mg. Thermogravimetric analysis was performed using a high-precision TGA/DSC1 system (Mettler-Toledo, Switzerland). Heating was performed from 25 °C to 950 °C at a heating rate of 20°C/min in an airflow of 50 mL/min. The percentage of weight loss at 25°C–200°C corresponds to water loss, 200°C–600°C to organic matter content loss (%OM), 600–950°C to carbonate loss (%CO_3_), and the remaining weight corresponds to phosphate content (%PO_4_).^26^ The weight loss percentage was calculated as: mass loss% = (Δm/m_0_) × 100, in which Δm is the mass loss within each temperature interval and m_0_ is the initial sample mass.

### Mechanical tests

Specimens from each group were subjected to three-point bending tests to measure mechanical properties. Specimen dimensions were verified using digital calipers (precision ±0.01 mm) and recorded. Only specimens with uniform rectangular cross-sections and dimensions within ±5% of target values (6.0 × 1.0 × 1.0 mm^3^) were included in the analysis. Mechanical testing was performed using a universal testing machine (Instron 3345 system, Instron Co., USA) equipped with a 500 N load cell, on a two-point support (with a length of 4.0 mm between points), at a loading speed of 0.5 mm/min until specimen fracture. Flexural strength (MPa) was estimated using the standard three-point bending formula for rectangular cross-sections: $\mathrm{MPa}=3PL/2wt^{2}$, in which *P* is the load at fracture, *L* is the distance between supports, *w* is the width, and *t* is the thickness of the beam.

### Statistical analysis

Sample size estimation was conducted using G*Power software (v.3.1.9.7; Heinrich-Heine-Universitat Düsseldorf, Düsseldorf, Germany) for flexural strength (primary outcome; ANOVA: fixed effects, omnibus, 4 groups): effect size f=0.4, α=0.05, 4 groups, yielding a minimum of n=9 per group. Mean and standard deviation were estimated for all measured parameters across experimental groups. Normality was assessed using the Shapiro-Wilk test. Non-parametric data were analyzed using the Kruskal-Wallis test followed by Mann-Whitney *U* pairwise comparisons. Analyses were performed using SPSS 24.0 (SPSS Inc., Chicago, USA) and OriginPro 2018 (OriginLab Corporation, Massachusetts, USA). Significance was set at p<0.05.

## Results

### Physicochemical characterization of NPs

**Figure 1:**
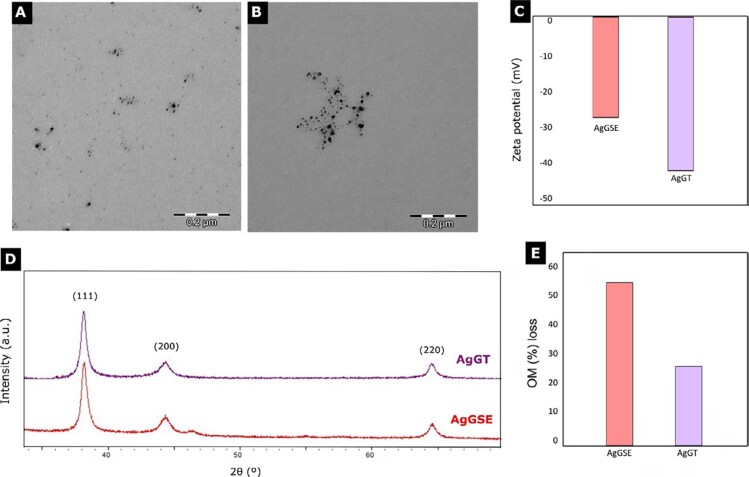
TEM micrographs of synthesized AgGSE (A) and AgGT (B) nanoparticles; scale bars: 0.2 μm. Surface zeta potential measurements of both NPs (C). XRD patterns of AgGT and AgGSE nanoparticles (D). TGA results showing the percentage of organic matter loss for both NPs (E).

TEM analysis revealed that both AgGSE and AgGT nanoparticles presented a homogeneous size distribution, with average diameters around 20 nm (Figures 1A and 1B). The nanoparticles exhibited predominantly spherical morphologies and a core-shell structure, with a high electron density metallic silver core and a low electron density organic shell, indicating efficient functionalization by the plant extract compounds, confirming successful surface coating. Surface zeta potential measurements at pH 6.5 showed that both nanoparticle types carried a negative charge, with AgGSE exhibiting a zeta potential of −28 mV and AgGT of −43 mV (Figure 1C). XRD patterns for both samples confirmed the exclusive precipitation of crystalline silver, with peaks corresponding to metallic silver, specifically, (111), (200), and (220) lattice planes (Figure 1D). The presence of well-defined but broadened diffraction peaks is consistent with the nanocrystalline nature of the materials. Crystallite size (D), estimated using Scherrer’s formula from the width of the prominent (111) reflection,^27^ yielded an average dimension of 21 nm for both AgGT and AgGSE, in agreement with TEM measurements. TGA revealed differences in organic content between the two nanoparticle types (Figure 1E). AgGSE NPs presented 50% (w/w) organic matter loss, which is double the value observed for AgGT NPs (20% w/w).

### Cytocompatibility of NPs

**Figure 2:**
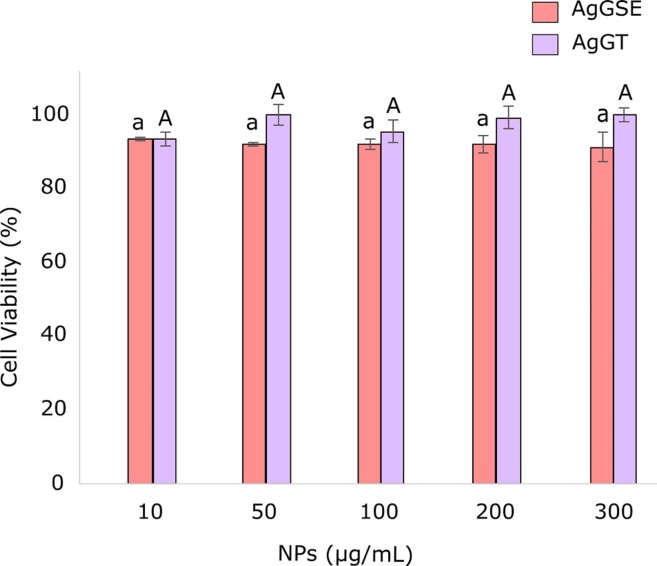
Cell viability of fibroblasts exposed to AgGSE and AgGT nanoparticles (10–300 μg/mL), determined by MTS assay. Data represent mean ± standard deviation. Groups sharing the same letter indicate no statistically significant differences (p>0.05).

The cytotoxicity of AgGSE and AgGT was evaluated over a broad concentration range (10–300 μg/mL) in fibroblast cultures using the MTS assay. As shown in Figure 2, both NPs maintained high cell viability across all concentrations, with measured values consistently ranging from 91% to 100%. Notably, even at the highest concentration (300 μg/mL), viability remained above 90%, with no statistically significant differences between groups (p≥0.10).

### Analyses of treated dentin samples

#### ATR-FTIR analyses

**Figure 3:**
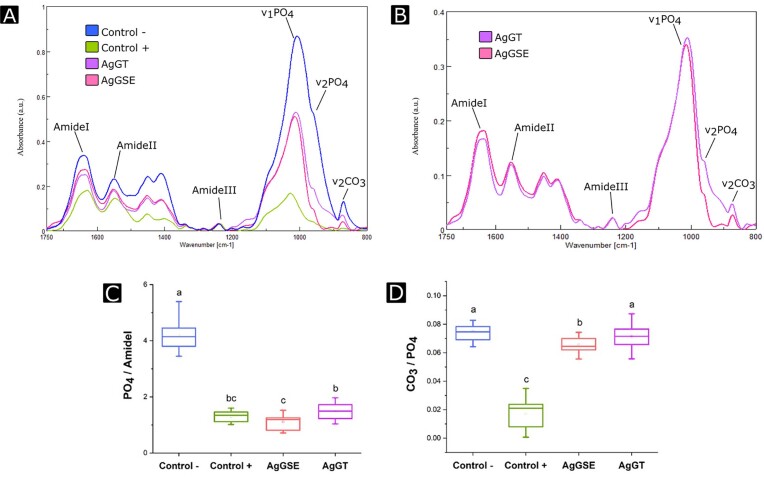
ATR-FTIR analysis of dentin chemical composition. (A) Representative spectra of all groups, normalized to the Amide III peak (~1240 cm-1). (B) Comparative spectra of AgGSE and AgGT, normalized to the Amide III peak (~1240 cm-1). (C) PO4/Amide I ratios. (D) CO3/PO4 ratios. Data represent mean ± SD. Different letters indicate statistically significant differences between groups (p<0.05).

The main results from ATR-FTIR analysis of dentin samples are presented in Figure 3. Data indicate that both pH-cycling and AgNP treatment induced significant alterations in chemical composition. The negative control group exhibited higher mineralization (PO_4_/Amide I ratio, p<0.001, Figure 3C) and more intense PO_4_ and CO_3_ peaks compared to all pH-cycled groups (Figure 3A). The positive control showed an Amide I band shift (~1640→1625 cm^-1^) and reduced PO_4_ intensity (Figure 3A), indicating demineralization. AgGT-treated dentin demonstrated superior remineralization (PO_4_/Amide I ratio, p=0.003; Figure 3C), higher carbonate substitution (CO_3_/PO_4_ ratio, p=0.03; Figure 3D), and more intense and broadened PO_4_ peaks compared to AgGSE (Figure 3B). Conversely, AgGSE-treated dentin presented increased Amide I and Amide II intensities (Figure 3B).

#### XRD analyses

The effects of different treatments on dentin crystallinity were assessed by XRD. Crystallite sizes (D) of dentin apatite, estimated from the 002-apatite reflection, are summarized in Table 1. Crystallite size in the negative control group was larger than in the AgNP-treated groups (p≤0.028), with the AgGSE group exhibiting the smallest average crystallite size (p≤0.047).

**Table 1:** Mean (standard deviation) values of full width at half maximum (FWHM) and crystallite size (D) from the 002 apatite peak.

Group	FWHM	D (nm)	
**Control -**	**0.419 (0.013)**	**23 (0.58)**	**a**
Control +	0.428 (0.013)	22 (0.58)	ab
AgGSE	0.538 (0.125)	18 (3.78)	c
AgGT	0.496 (0.071)	19 (2.49)	bc

Different letters indicate statistically significant differences (Kruskal-Wallis test followed by Mann-Whitney U pairwise comparisons, p<0.05).

#### TGA measurements

**Figure 4:**
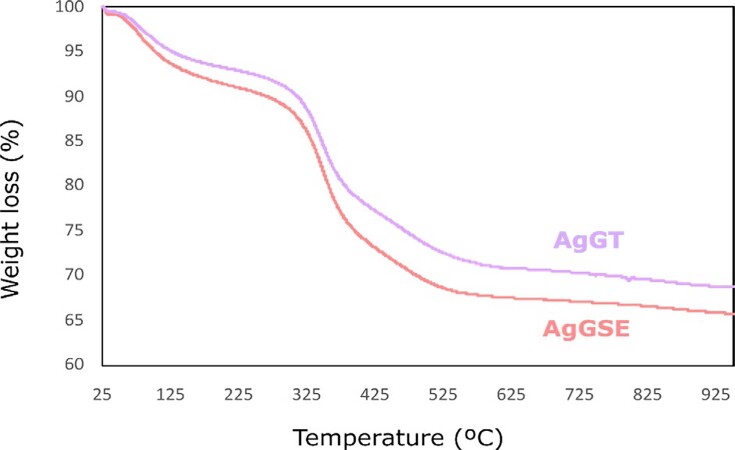
TGA curves showing weight loss (%) as a function of temperature for dentin samples treated with AgGT and AgGSE nanoparticles.

Thermogravimetric analysis results, summarized in Table 2, confirmed that the negative control group exhibited the highest dentin mineral content, as indicated by %PO_4_ (p≤0.049). AgGT-treated dentin demonstrated a higher CO_3_/PO_4_ ratio (p=0.007) compared to AgGSE group. Figure 4 presents the TGA curves for dentin treated with AgGT and AgGSE. Both groups exhibited three distinct weight loss events: the first between 25 °C and 120 °C (water evaporation); and the second and third between 120 °C and 500 °C (combustion of organic matter and catechins). At 500 °C, AgGSE-treated specimens showed a total mass loss of ~32%, whereas AgGT-treated samples displayed ~28%.

**Table 2:** Mean (standard deviation) weight loss percentages of water, organic matter (OM), CO3, and PO4 from TGA analysis.

Group	Water (%)	OM (%)	CO_3_ (%)	PO_4_ (%)	CO_3_/PO_4_	PO_4_/OM
**Control -**	**6.81 (1.33)^a^**	**17.47 (2.98)^b^**	**2.32 (0.12)^a^**	**73.39 (4.31)^a^**	**0.32 (0.03)^ab^**	**4.32 (1.08)^a^**
Control +	7.21 (0.51)^a^	22.63 (1.37)^ab^	2.09 (0.17)^ab^	68.07 (1.63)^bc^	0.31 (0.02)^ab^	3.02 (0.25)^b^
AgGSE	7.26 (0.62)^a^	22.88 (2.54)^a^	1.84 (0.16)^b^	68.02 (3.13)^b^	0.27 (0.04)^b^	3.01 (0.43)^b^
AgGT	7.28 (0.31)^a^	21.23 (2.78)^ab^	2.78 (1.17)^a^	68.71 (1.88)^ab^	0.40 (0.16)^a^	3.30 (0.55)^ab^

Estimated ratios of CO_3_/PO_4_ and PO_4_/OM are presented. Different letters indicate statistically significant differences (Kruskal-Wallis test followed by Mann-Whitney U pairwise comparisons, p<0.05).

#### Mechanical properties

**Figure 5:**
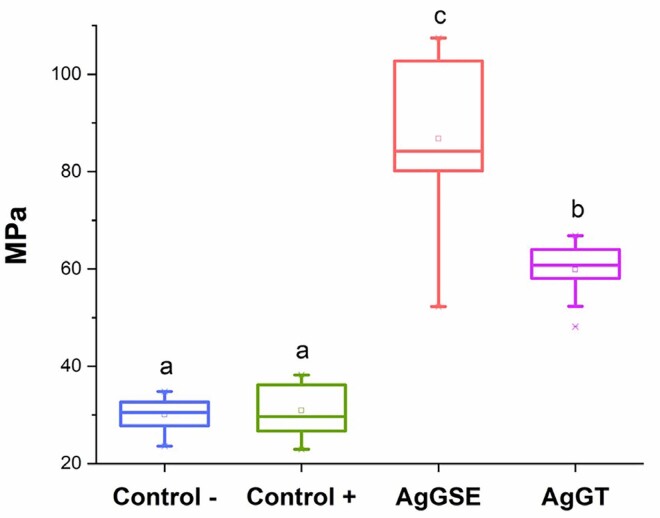
Flexural strength (MPa) of dentin specimens determined by three-point bending test. Different letters indicate statistically significant differences between groups (p<0.05).

The results of the three-point bending tests (Figure 5) revealed significant differences in dentin mechanical properties between groups. The control groups exhibited significantly lower flexural strength compared to the NP-treated groups (p<0.001). Among treated groups, AgGSE showed the highest flexural strength, significantly greater than all other groups (p≤0.001).

## Discussion

In this study, we produced and characterized AgNPs using green chemistry, with plant extracts rich in natural cross-linking polyphenols as reducing and stabilizing agents. This is the first study to compare their characteristics and behavior on demineralized dentin for potential clinical use. Both synthesized AgNPs were biocompatible and showed promising activity, although they exhibited different behaviors on demineralized dentin. AgGSE NPs exhibited higher surface organic content (Figure 1E), and AgGSE-treated samples showed increased flexural strength (Figure 5) and smaller apatite crystallite sizes (Table 1). In contrast, AgGT-treated dentin presented higher PO_4_/Amide I and CO_3_/PO_4_ ratios (Figures 3B,C; Table 2).

Polyphenols such as catechins donate electrons and reduce silver ions (Ag^+^) to metallic silver (Ag^0^) because the B-ring hydroquinone is oxidized to semiquinone or quinone, which darkens the solution.^14,18,28^ Therefore, color darkening (Figure S1) provided the first visual indication of NP formation.^18,29^ After biosynthesis, both NPs exhibited very similar morphology and size distribution (Figures 1A,B), microstructural characteristics (Figure 1D), and surface chemistry properties (Figure 1C). XRD confirmed the formation of silver nanoparticles in both groups (Figure 1D).^17,18^ TGA revealed higher organic content in AgGSE particles than in AgGT (Figure 1E). TEM images (Figures 1A, B) show the spherical shape and core-shell structure of both particles. The organic-rich shell is attributed to phenolic compounds coating the particles, whereas the core is metallic silver.^14^ This core-shell structure provides further evidence of effective formation of cross-linker-functionalized AgNPs. NP stability in aqueous suspension can be quantified by the surface zeta potential due to the repulsion and attraction forces between particles.^17^ A high zeta potential (≥25 mV), negative or positive, indicates strong repulsion, resulting in dispersion and stability of the NPs suspension.^17^ Zeta potential measurements indicated notable colloidal stability for both nanoparticles, with AgGT exhibiting higher stability (Figure 1C). The negative charge confirms polyphenol adsorption on the nanoparticle surface.^17,18^ FTIR analysis shows AgGSE intensified Amide I/II bands indicate a thicker, sterically hindered organic capping layer from proanthocyanidin-collagen cross-linking, reducing surface charge density and electrostatic repulsion.^30,31^ In contrast, lower organic content reflects optimized monolayer capping with enhanced catechin OH ionization.^32^ TGA showed weight loss from 200°C to 600°C corresponding to organic matter content (%OM).^26^Figure 1E shows that AgGSE had approximately twice the %OM of AgGT, indicating higher phenolic content. Based on these findings, both NPs were effectively synthesized, with AgGSE showing higher phenolic content (TGA, Figure 1E) and AgGT greater stability (zeta potential, Figure 1C). Accordingly, the first null hypothesis was rejected because NPs differ in physicochemical characteristics.

AgNP high cytotoxicity is related to the induction of reactive oxygen species (ROS) and DNA damage.^33,34^ Polyphenols are antioxidants that inhibit ROS and protect cell surfaces.^35^ As shown in Figure 1, the polyphenolic coating acts as a protective barrier that scavenges ROS, limits Ag^+^ ion release, and shields cell membranes from direct NP contact, thereby reducing cytotoxicity.^36-38^ Therefore, the antioxidant properties of GSE and GT in AgNPs improve biocompatibility of NPs.^17,35^ Although studies have shown that both AgGSE and AgGT are non-toxic even at high concentrations (i.e., 100 μg/mL), the cell line used must be considered because different cell lines demonstrate different biocompatibility rates.^17,35^ Furthermore, the cytotoxicity of AgNPs also depends on other parameters such as particle size, surface area, and surface reactivity.^33^ Our research follows the ISO 10993-5 guidelines for the biological evaluation of medical devices to ensure their safety and biocompatibility. According to these standards, materials with cell viability of 70% or higher are considered non-cytotoxic. Cytotoxicity assays using 3T3 fibroblasts demonstrated that AgGSE and AgGT maintained high viability (91%–100%, Figure 2), confirming that both NPs are non-toxic regardless of concentration (p≥0.10). Therefore, the second null hypothesis was rejected because AgGSE and AgGT are biocompatible nanoparticles.

Demineralization/remineralization experiments were validated by complementary ATR-FTIR and XRD analyses. Negative control showed higher mineralization (PO_4_/Amide I ratio, p<0.001; intense PO_4_ peak) and crystallinity (XRD, p≤0.028) compared to pH-cycled groups (Figures 3A,C; Table 1), confirming pH-cycling efficacy.^39^ Conversely, positive control showed Amide I band shift and reduced PO_4_ intensity, consistent with demineralization-induced collagen structural changes (Figure 3A).^40^ The elevated CO_3_/PO_4_ ratio (p<0.001, Figure 3D) observed in the negative control reflects natural mature dentine chemistry with type B carbonate substitutions in hydroxyapatite.^41,42^ In contrast, the positive control had the lowest ratio (p<0.001), indicating significant mineral loss due to pH-cycling. Both nanoparticle-treated groups demonstrated higher CO_3_/PO_4_ ratios compared to the positive control (p<0.001), indicating carbonated apatite deposition characteristic of partial remineralization through type B carbonate incorporation. AgGT-treated dentin showed a CO_3_/PO_4_ ratio closely approximating that of natural dentin (p=0.272), while AgGSE-treated samples exhibited significantly lower ratios (p=0.033). Furthermore, AgGT demonstrated a higher PO_4_/Amide I ratio than AgGSE (p=0.003, Figure 3C). Confirming these findings, the AgGT spectra exhibited notable broadening and increased intensity of the main phosphate (ν_1_, ν_3_ PO_4_; 900-1200 cm^-1^) and the carbonate ν_2_ band (~870 cm^1^) (Figure 3B), suggesting the deposition of a biologically relevant carbonate-enriched mineral phase that mimics physiological mineralization patterns. Conversely, AgGSE treatment favors a less carbonated mineral phase associated with higher phenolic content (Figure 1E, Figure 3B, and Figure 4) and enhanced mechanical properties (Figure 5). Correspondingly, FTIR spectra revealed that Amide I (~1630 cm^-1^, primarily C=O stretching) and Amide II (~1550 cm^-1^, N-H bending coupled to C-N stretching) peaks showed broadening and increased intensity in AgGSE-treated dentin (Figure 3B), indicating proanthocyanidin-mediated collagen cross-linking that stabilizes the triple helix and introduces conformal heterogeneity.^40,43,44^ These complementary FTIR findings demonstrate that AgGSE produces a strongly biomodified collagen scaffold that guides apatite crystallization, explaining its superior flexural strength (Figure 5) despite lower mineral content. They also confirm distinct remineralization pathways: organic-assisted crystal stabilization by AgGSE and biomimetic mineral deposition by AgGT.^10,43,45,46^ Recent nanoparticle strategies reinforce this paradigm: Liu, et al.^47^ (2025) demonstrated Mn-doped whitlockite NPs enhancing bone regeneration via osteogenic and mechanical reinforcement, paralleling AgNPs’ dentin matrix stabilization. Yang, et al.^48^ (2024) reviewed ACP stabilizers for biomimetic dentin remineralization, contextualizing polyphenol-AgNPs as complementary approaches to mineral deposition and organic-inorganic synergy.

XRD analysis of dentin samples revealed higher crystallinity in the negative control group (larger apatite crystallite size, p≤0.028; Table 1), consistent with the elevated PO_4_/Amide ratio (p<0.001) and intense PO_4_ peak observed by ATR-FTIR (Figures 3A,C), reflecting more mature, well-ordered apatite crystals. In contrast, the experimental groups presented the lowest crystallinity (p≤0.047, Table 1), which may be attributed to the formation of smaller and less ordered apatite crystals or a greater proportion of amorphous mineral phases typical of newly deposited apatite.^49^ These findings suggest that nanoparticle treatments induce remineralization characterized by the deposition of early-stage mineral phases with distinct structural features compared to native dentin. Moreover, while high crystallinity generally indicates mineral maturity, it can also increase brittleness and reduce the tissue’s capacity to withstand flexural stress, potentially explaining the lower flexural strength observed in control groups (Figure 5).

The phenolic hydroxyl groups of tannins interact with the carbonyl amide groups of dentin collagen through covalent, ionic, hydrogen, and hydrophobic bonds.^9,50^ The bioactivity of these extracts depends greatly on their structural complexity, chemical composition, and extraction process.^9,51^ GT from *Camellia sinensis* contains large amounts of EGCG, whereas GSE from *Vitis vinifera* predominantly contains non-galloylated oligomers. Although both extracts are capable of interacting with the dentin matrix via chemical bonds, differences in monomer content, degree of polymerization, and galloylation result in distinct interactions with organic matter.^51,52^ Notably, GSE exhibits a stronger and more stable association with the dentin matrix than GT, as excess galloyl groups can reduce biomodification potential.^53^ TGA revealed a higher organic fraction in AgGSE-treated dentin (32% weight loss at 500 °C) versus AgGT (28%) (Figure 4), consistent with greater proanthocyanidin incorporation. The increased cross-linking of the organic matrix correlates with superior flexural strength (p<0.001, Figure 5), highlighting the collagen matrix’s critical role in dentin biomechanics. Additionally, TGA confirmed a higher CO_3_/PO_4_ ratio in AgGT-treated dentin relative to AgGSE (p=0.007, Table 2), corroborating the findings obtained through ATR-FTIR analysis. Previous studies have shown that AgNPs are able to infiltrate the dentin matrix, bind to hydroxyapatite, and form insoluble silver chloride, thereby increasing mineral density.^54,55^ Furthermore, topical application of AgNO_3_ to demineralized dentin is capable of forming a protective Ag_3_PO_4_ barrier as a result of interactions between Ag^+^ ions and PO_4_^3-^ of hydroxyapatite, contributing to increased mineral density.^21^ Given these findings, we suggest that AgGT delivers a greater proportion of free Ag^+^ ions compared to AgGSE, which promotes further reduction of Ag^+^ ions. Consequently, the greater availability of Ag^+^ ions in AgGT may facilitate enhanced mineral formation within the dentin, as these ions can directly interact with the PO_4_^3-^ groups of hydroxyapatite.

The mechanical properties of dentin result from the synergy between organic and inorganic phases.^10^ While mineral content provides rigidity, the collagen matrix contributes toughness and elasticity.^56^ Specifically, dentinal tubule-collagen interactions are responsible for the elastic properties of dentin.^56,57^ Three-point bending tests revealed that both AgNPs significantly increased flexural strength compared to controls (p<0.001, Figure 5). However, despite AgGT higher mineral content (Figures 3B,C,D; Table 2), AgGSE achieved superior flexural strength (p≤0.001, Figure 5). This is attributed to the higher proanthocyanidin content of AGSE (Figure 1E), which promotes collagen cross-linking (broadened Amide I/II bands, Figure 3B) and triple helix stabilization^43^, thus reinforcing mechanical resistance. The data suggest that organic phase reinforcement has a predominant influence on the flexural performance of dentin after treatment, emphasizing the importance of integrating organic and mineral stabilization strategies in restorative material design. It is also noteworthy that AgGSE exhibited high standard deviations in flexural strength (Figure 5), a variance attributable to the greater amorphous content, lower crystallinity, and reduced chemical stability compared to AgGT, as evidenced by XRD, TGA, and zeta potential measurements. Importantly, this variability did not compromise the superior mechanical performance. Since optimal mechanical performance is a core criterion for dental biomaterials, these findings support the conclusion that both NPs effectively reinforce dentin, with AgGSE displaying especially promising results.

Previous studies have demonstrated AgNPs’ capacity to promote enamel remineralization mainly through surface mineral precipitation, increased microhardness, and partial restoration of the Ca/P ratio on demineralized enamel without directly addressing collagen stabilization.^58-60^ However, their behavior in dentin is inherently more complex due to the higher organic content and tubular microstructure of this substrate. Our dentin model revealed distinct, substrate-dependent effects: AgGSE-functionalized nanoparticles primarily enhanced biomechanical reinforcement likely through polyphenol-mediated collagen cross-linking, whereas AgGT-functionalized nanoparticles more closely mimicked the enamel pattern, promoting higher mineral deposition and CO_3_/PO_4_ reorganization. These findings suggest that, although AgNP-based systems can support remineralization in both enamel and dentin, optimal formulations should be tailored to the specific microenvironment, prioritizing organic matrix stabilization in dentin and surface mineral reconstruction in enamel.

The 1-minute treatment duration was selected to promote adequate diffusion and interaction of the AgNPs formulations within dentin beams (1 mm thickness). For instance, Wang et al.^40^ (2021) used 30 seconds for polyphenol-rich extracts on demineralized dentin films (10 μm thick), achieving rapid inhibition of gelatinolytic activity due to their minimal thickness. Similarly, Nisar et al.^61^ (2023) applied dual-functional etchants with crosslinkers for 30 seconds to ultrathin dentin sections (5 μm thick), relying on acid-mediated diffusion for collagen stabilization. However, given the greater thickness of the dentin beams in this study, extending the treatment to 1 minute ensured uniform penetration for both AgNPs types, enabling reliable detection of comparative differences in remineralization efficacy, as evidenced by our FTIR, XRD, TGA, and mechanical tests.

Although the antimicrobial properties of silver nanoparticles are well established, this study represents the first comprehensive comparison of AgNPs synthesized with distinct natural polyphenols (GSE and GT) on demineralized dentin, addressing critical gaps in understanding their mechanisms of action and clinical potential. Using sustainable green chemistry, we produced biocompatible AgNPs with polyphenols as reducing and stabilizing agents. Beyond safety, short-term topical application (1 min) effectively reinforced mechanical properties and promoted remineralization, positioning both formulations as promising agents for preventive and caries-control therapy. Comparative analysis revealed distinct effects: AgGSE enhanced flexural strength (Figure 5) via proanthocyanidin-mediated collagen cross-linking (Figure 3B; Table 2), whereas AgGT promoted biological remineralization through carbonated apatite deposition (Figures 3B,C,D; Table 2). These complementary pathways highlight green-synthesized AgNPs as multifunctional biomaterials that restore both structural integrity and mineralization to compromised dentin.

Hence, the third null hypothesis was rejected because AgGSE and AgGT produce different effects on the mineral composition (FTIR, Figures 3B,C,D; TGA, Table 2) and biomechanical properties of demineralized dentin (Figure 5). Study limitations include the *in vitro* design (limiting clinical translation despite human dentin use), short-term three-point bending assessment, and absence of biofilm challenge. Additional research, including *in vivo* studies and long-term biocompatibility/performance assessments in the oral environment, is essential to fully elucidate and differentiate the mechanisms and clinical potential of these nanoparticles.

## Conclusion

Silver nanoparticles were effectively synthesized by green chemistry using *Camellia sinensis* leaf extract and *Vitis vinifera* grape seed extract as reducing and stabilizing agents. Both NPs demonstrated biocompatibility and possessed crystalline, spherical silver nanostructures, with differences in organic content and thermal stability. Short-term application (1 minute) to demineralized dentin improved mechanical properties in all treated specimens. Notably, AgGSE-treated dentin exhibited higher flexural strength, whereas AgGT showed greater remineralization potential as indicated by the PO_4_/Amide I and CO_3_/PO_4_ ratios. These findings suggest that green-synthesized silver nanoparticles are suitable alternatives for the treatment of demineralized dentin.

## Supplementary Figure

**Supplementary Figure S1:**
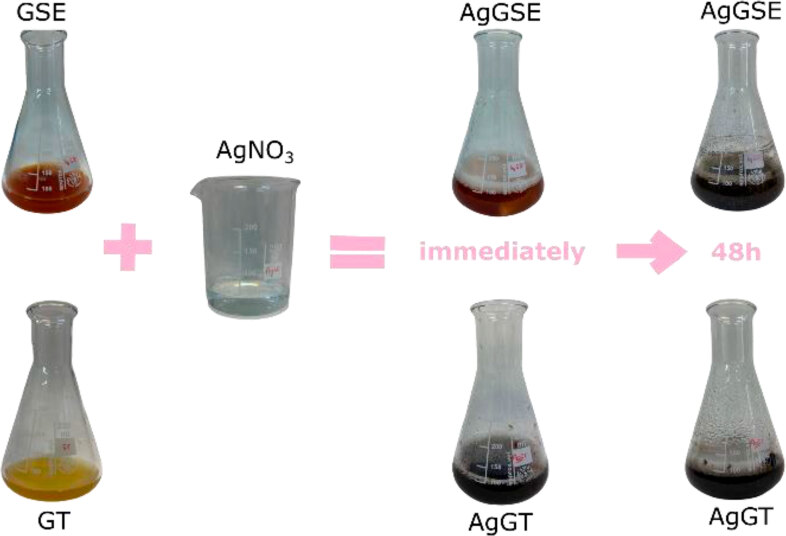
- Photographic documentation of green synthesis progression. Upper panels: Light brown extract (GSE) -> dark brown (GSE + AgNO_3_ immediate) -> brown-gray (AgGSE 48h). Lower panels: Dark yellow extract (GT) -> yellow-gray (GT + AgNO_3_ immediate) -> gray-black (AgGT 48h). Darkening confirms polyphenol-mediated Ag^+^ reduction to metallic AgNPs.
